# Supplementary material for: Delphi Method to Achieve Clinical Consensus for a BPMN Representation of the Central Venous Access Placement for Training Purposes
Source: Int J Environ Res Public Health. 2020 May 30;17(11):3889. doi: 10.3390/ijerph17113889 (PMC7312914; doi:10.3390/ijerph17113889)
Supplement: Supplementary file 1 [file Supplementary Figure S2.pdf › Supplementary Figure S2.pdf]

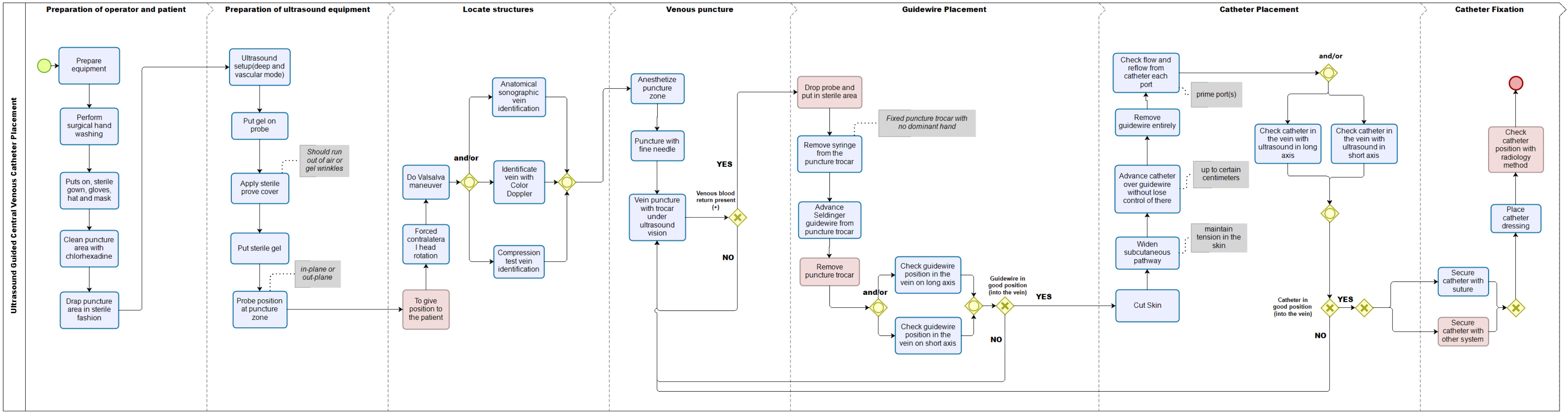

Supplementary Figure S2. Model of the Central Venous Access Placement with Ultrasonography used in Second Round of the Delphi Panel, represented in BPMN. Activities that were suggested by the experts after the first survey are displayed in red.
