# Supplementary material for: Delphi Method to Achieve Clinical Consensus for a BPMN Representation of the Central Venous Access Placement for Training Purposes
Source: Int J Environ Res Public Health. 2020 May 30;17(11):3889. doi: 10.3390/ijerph17113889 (PMC7312914; doi:10.3390/ijerph17113889)
Supplement: Supplementary file 1 [file ijerph-17-03889-s001.zip › Supplementary Document S4.pdf]

The following is a new diagram of the installation process of a central venous catheter which the items on the next page are based on.

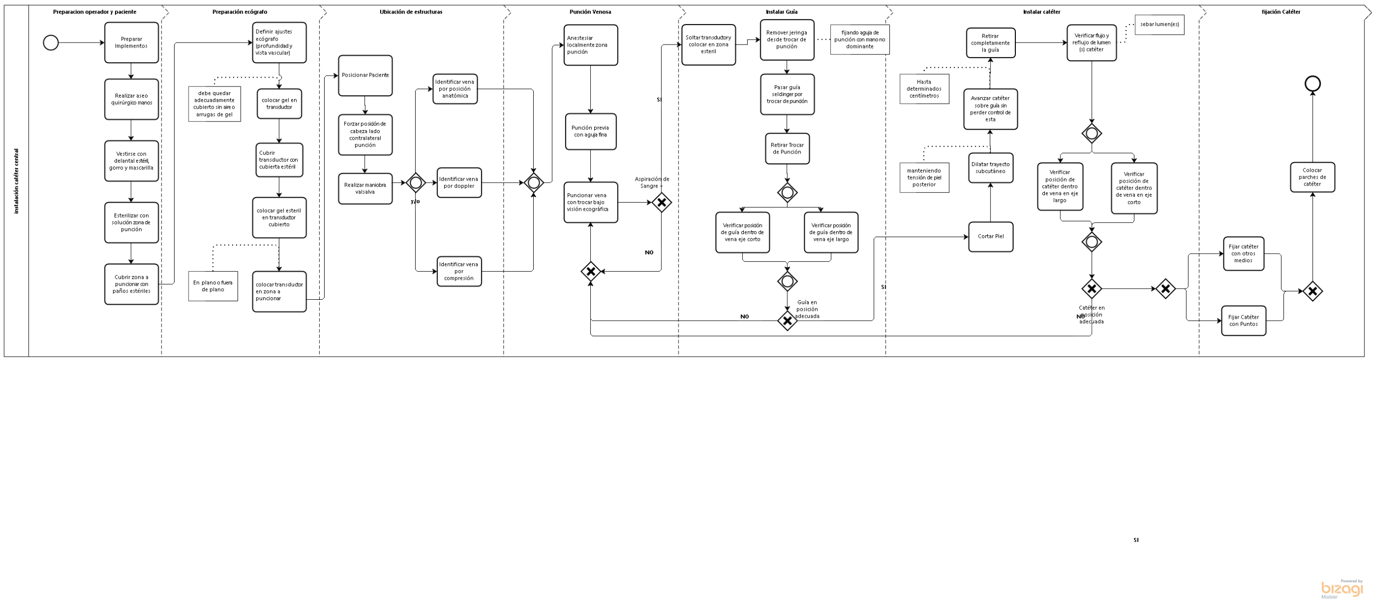

By clicking on the following link you can gain direct access to the image of the proposed model, which can be printed if so desired.

[Model Version 2 "Central Venous Access Placement"]

Below we will only request your name to identify your responses.

* 1. Name

Sig.

Con la tecnología de

The model previously seen in a diagram is given below as a series of sequential items which are activities suggested as possible steps in said process.

First survey results:
The percentages of responses for the items on the Likert scale that were given in the previous round of the Delphi panel are given in brackets, according to the following specification:

Red colour: percentage of responses “Under no circumstances should be included” plus “Should not be included”
Black colour: percentage of responses "May or may not be included"
Green Colour: percentage of responses “Should be included” plus “Must be included”

Example: Activity xxxxxxxxx
(red - black - green)

Activities suggested in the first survey:

The activities that were suggested by you in the first round have been added to the list without an identifying number and no percentage of responses. In some cases the suggestions have enriched an already defined activity. The new information has been added to the activity in italics.

How to respond this time:
Again for each item, say how sure you are that the activity should be included in a generic process model according to the given scale

* 2. Encuesta:

	Under no circumstances should be included	Should not be included	May or may not be included	Should be included	Must be included
1.-Prepare equipment<br>(0 - 8 - 92)	○	○	○	○	○
2.-Perform surgical hand washing<br>(0 - 0 - 100)	○	○	○	○	○
3.-Puts on, sterile gown, gloves, hat and mask<br>(0 - 0 - 100)	○	○	○	○	○
4.-Clean puncture area with chlorhexidine<br>(0 - 0 - 100)	○	○	○	○	○
5.-Drape puncture area in sterile fashion<br>(0 - 0 - 100)	○	○	○	○	○
6.-Ultrasound setup (deep and vascular mode)<br>(0 - 17 - 83)	○	○	○	○	○
7.-Put gel on probe<br>(8 - 8 - 84)	○	○	○	○	○
8.-Apply ***sterile*** probe cover<br>(0 - 0 - 100)	○	○	○	○	○
9.-Put sterile gel on covered transducer<br>(17 - 33 - 50)	○	○	○	○	○
10.-Probe position at puncture zone<br>(0 - 8 - 92)	○	○	○	○	○
To give position to the patient	○	○	○	○	○
11.-Turn head away from puncture site<br>(17 - 58 - 25)	○	○	○	○	○
12.-Do Valsalva maneuver<br>(25 - 58 - 17)	○	○	○	○	○
13.-Anatomical sonographic vein identification<br>(8 - 33 - 58)	○	○	○	○	○
14.-Identify vein with color Doppler<br>(0 - 50 - 50)	○	○	○	○	○
15.-Compression test vein identification<br>(0 - 33 - 67)	○	○	○	○	○
16.-Anesthetize puncture site<br>(0 - 25 - 75)	○	○	○	○	○
17.-Puncture with fine needle<br>(42 - 33 - 25)	○	○	○	○	○
18.-Vein puncture with trocar under ultrasound vision<br>(0 - 8 - 92)	○	○	○	○	○
19.-Venous blood return present (+)<br>(0 - 16 - 84)	○	○	○	○	○
Set probe in sterile area	○	○	○	○	○
20.-Remove syringe from the puncture trocar<br>(0 - 25 - 75)	○	○	○	○	○
21.-Advance Seldinger guidewire through puncture trocar<br>(0 - 8 - 92)	○	○	○	○	○
Remove puncture trocar	○	○	○	○	○
22.-Verification of the guide with ultrasound in long axis<br>(0 - 17 - 83)	○	○	○	○	○
23.-Verification of the guide with ultrasonography in short axis<br>(0 - 33 - 67)	○	○	○	○	○
24.-Guidewire in good position (in the vein)<br>(0 - 0 - 100)	○	○	○	○	○
25.-Cut skin<br>(25 - 58 - 17)	○	○	○	○	○
26.-Widen subcutaneous pathway<br>(0 - 8 - 92)	○	○	○	○	○
27.-Advance catheter over guidewire without losing control<br>(0 - 0 - 100)	○	○	○	○	○
28.-Remove guidewire entirely<br>(0 - 0 - 100)	○	○	○	○	○
29.-Check flow and reflow in each catheter port<br>(0 - 8 - 92)	○	○	○	○	○
30.-Check catheter in the vein with ultrasound in long axis<br>(0 - 42 - 58)	○	○	○	○	○
31.-Check catheter in the vein with ultrasound in short axis<br>(8 - 42 - 50)	○	○	○	○	○
32.-Catheter in good position (in the vein)<br>(0 - 8 - 92)	○	○	○	○	○
33.-Secure catheter with suture<br>(8 - 0 - 92)	○	○	○	○	○
Secure catheter with other systems	○	○	○	○	○
34.-Place catheter dressing<br>(0 - 8 - 92)	○	○	○	○	○
Check catheter position with radiology method	○	○	○	○	○

Ant. Sig.

3. . Would you add any other activities? Where would you locate it/them on the list? (e.g. Checking of Seldinger guideline between activities 6 and 7.)

1.- Activity:

2.- Activity:

3.- Activity:

Thank you very much for participating in our research.

Ant. Listo

Con la tecnología de
SurveyMonkey®
Ve lo fácil que es crear una encuesta.
