# Supplementary material for: Delphi Method to Achieve Clinical Consensus for a BPMN Representation of the Central Venous Access Placement for Training Purposes
Source: Int J Environ Res Public Health. 2020 May 30;17(11):3889. doi: 10.3390/ijerph17113889 (PMC7312914; doi:10.3390/ijerph17113889)
Supplement: Supplementary file 1 [file ijerph-17-03889-s001.zip › Supplementary Document S3.pdf]

The following is a diagram of the central venous catheter installation process, which the items on the following sheet are based on:

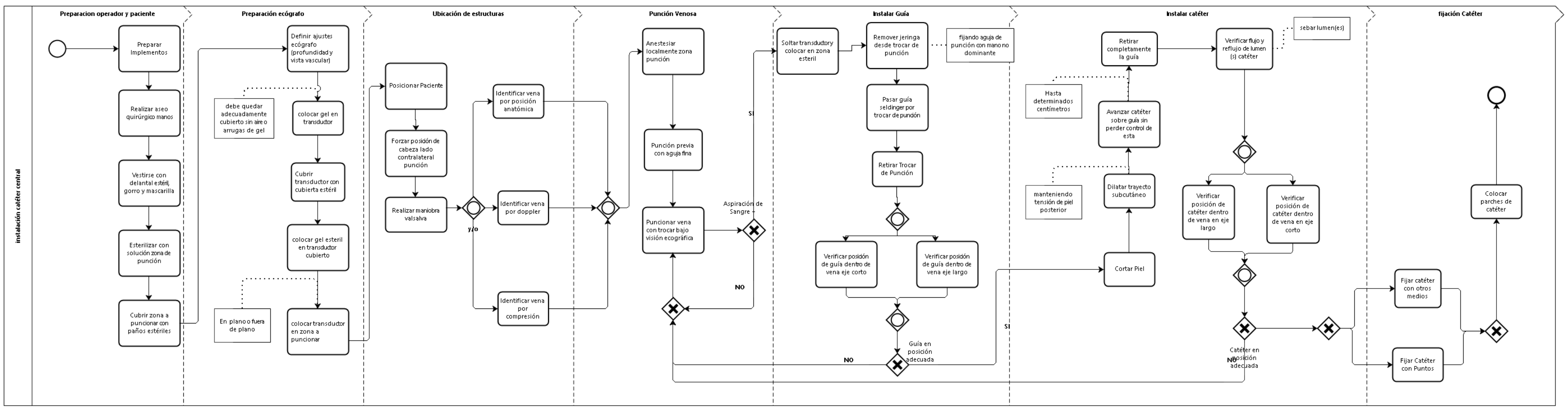

By clicking on the following link you can gain direct access to the image of the proposed model, which can be printed if so desired.

[**Central Venous Access Placement Process Model**]

Below we will request some basic information that will allow us to characterize the group of experts that make up our Delphi panel.

* 1. Name

2. Medical Specialty

3. Work Unit or Area (Operating Room , ICU, intermediate care unit, nephrology, other)

4. Contact E-mails

Sig.

Con la tecnología de
SurveyMonkey®
Ve lo fácil que es crear una encuesta.

The model previously seen in a diagram is given below as a series of sequential items which are activities suggested as possible steps in said process. Each one has an identifying number that corresponds to the place proposed in the model, and a name that describes the action involved and what it is done on or what it is done with.

* 5. For each item, say how sure you are that the activity should be included in a generic process model according to the given scale.

	Under no circumstances should be included	Should not be included	May or may not be included	Should be included	Must be included
**1.-Prepare equipment**	○	○	○	○	○
**2.-Perform surgical hand washing**	○	○	○	○	○
**3.-Puts on, sterile gown, gloves, hat and mask**	○	○	○	○	○
**4.-Clean puncture area with chlorhexidine**	○	○	○	○	○
**5.-Drape puncture area in sterile fashion**	○	○	○	○	○
**6.-Ultrasound setup (deep and vascular mode)**	○	○	○	○	○
**7.-Put gel on probe**	○	○	○	○	○
**8.-Apply sterile probe cover**	○	○	○	○	○
**9.-Put sterile gel on covered transducer**	○	○	○	○	○
**10.-Probe position at puncture zone**	○	○	○	○	○
**11.-Turn head away from puncture site**	○	○	○	○	○
**12.-Do Valsalva maneuver**	○	○	○	○	○
**13.-Anatomical sonographic vein identification**	○	○	○	○	○
**14.Identify vein with color Doppler**	○	○	○	○	○
**15.-Compression test vein identification**	○	○	○	○	○
**16.-Anesthetize puncture site**	○	○	○	○	○
**17.-Puncture with fine needle**	○	○	○	○	○
**18.-Vein puncture with trocar under ultrasound vision**	○	○	○	○	○
**19.-Venous blood return present (+)**	○	○	○	○	○
**20.-Remove syringe from the puncture trocar**	○	○	○	○	○
**21.-Advance Seldinger guidewire through puncture trocar**	○	○	○	○	○
**22.-Verification of the guide with ultrasound in long axis**	○	○	○	○	○
**23.-Verification of the guide with ultrasonography in short axis**	○	○	○	○	○
**24.-Guidewire in good position (in the vein)**	○	○	○	○	○
**25.-Cut skin**	○	○	○	○	○
**26.Widen subcutaneous pathway**	○	○	○	○	○
**27.-Advance catheter over guidewire without losing control**	○	○	○	○	○
**28.-Remove guidewire entirely**	○	○	○	○	○
**29.-Check flow and reflow in each catheter port**	○	○	○	○	○
**30.Check catheter in the vein with ultrasound in long axis**	○	○	○	○	○
**31.-Check catheter in the vein with ultrasound in short axis**	○	○	○	○	○
**32.-Catheter in good position (in the vein)**	○	○	○	○	○
**33.-Secure catheter with suture**	○	○	○	○	○
**34.Place catheter dressing**	○	○	○	○	○

Ant. Sig.

Con la tecnología de
SurveyMonkey®
Ve lo fácil que es crear una encuesta.

6. Would you add any other activities? Where would you locate it/them on the list? (e.g. Checking of Seldinger guideline between activities 6 and 7.)

1.- Activity:

2.- Activity:

3.- Activity:

4.- Activity:

5.- Activity:

6.- Activity:

7.- Activity:

8.- Activity:

7. Would you put forward a colleague who has experience in catheter installations with ultrasound that could contribute to this research by answering this survey? If so, please give his/her name and email or telephone number to contact him/her.

Nombre

E-mail Address

Telephone Number

Thank you very much for participating in our research. When we finish gathering the responses from the rest of our colleagues we will consolidate the information and share a second version of the model with you.

Ant. Listo
