# Supplementary material for: Delphi Method to Achieve Clinical Consensus for a BPMN Representation of the Central Venous Access Placement for Training Purposes
Source: Int J Environ Res Public Health. 2020 May 30;17(11):3889. doi: 10.3390/ijerph17113889 (PMC7312914; doi:10.3390/ijerph17113889)
Supplement: Supplementary file 1 [file ijerph-17-03889-s001.zip › Supplementary Figure S1.pdf]

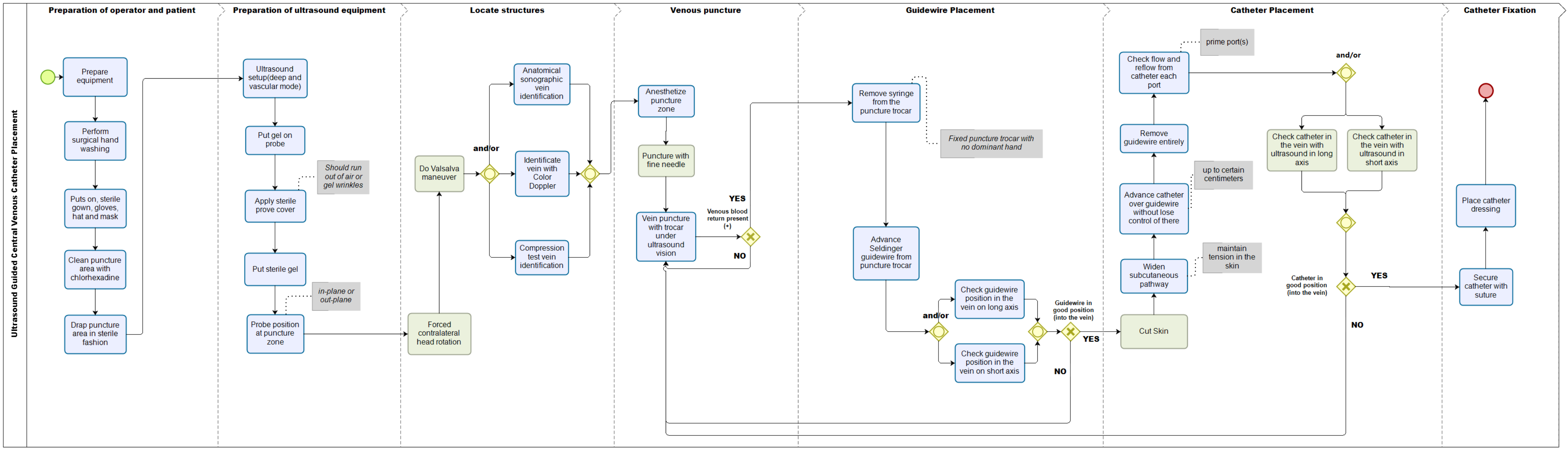

Supplementary Figure S1. Model of the Central Venous Access Placement with Ultrasonography used in First Round of the Delphi Panel, represented in BPMN. Activities that were not included in the final model are displayed in green.
